# Modeling and Applying Implicit Dormant Features for Recommendation via Clustering and Deep Factorization

**DOI:** 10.3390/s22218224

**Published:** 2022-10-27

**Authors:** Alpamis Kutlimuratov, Akmalbek Bobomirzaevich Abdusalomov, Rashid Oteniyazov, Sanjar Mirzakhalilov, Taeg Keun Whangbo

**Affiliations:** 1 Department of Computer Engineering, Gachon University, Sujeong-gu, Seongnam-si 461-701, Korea; 2 Department of Telecommunication Engineering, Nukus Branch of Tashkent University of Information Technologies Named after Muhammad Al-Khwarizmi, Nukus 230100, Uzbekistan; 3 Department of Information-Computer Technologies and Programming, Tashkent University of Information Technologies Named after Muhammad Al-Khwarizmi, Tashkent 100200, Uzbekistan

**Keywords:** recommendation system, clustering-based recommendation system, heterogeneous information, weighted nonnegative matrix factorization, implicit features, tag information, deep factorization

## Abstract

E-commerce systems experience poor quality of performance when the number of records in the customer database increases due to the gradual growth of customers and products. Applying implicit hidden features into the recommender system (RS) plays an important role in enhancing its performance due to the original dataset’s sparseness. In particular, we can comprehend the relationship between products and customers by analyzing the hierarchically expressed hidden implicit features of them. Furthermore, the effectiveness of rating prediction and system customization increases when the customer-added tag information is combined with hierarchically structured hidden implicit features. For these reasons, we concentrate on early grouping of comparable customers using the clustering technique as a first step, and then, we further enhance the efficacy of recommendations by obtaining implicit hidden features and combining them via customer’s tag information, which regularizes the deep-factorization procedure. The idea behind the proposed method was to cluster customers early via a customer rating matrix and deeply factorize a basic WNMF (weighted nonnegative matrix factorization) model to generate customers preference’s hierarchically structured hidden implicit features and product characteristics in each cluster, which reveals a deep relationship between them and regularizes the prediction procedure via an auxiliary parameter (tag information). The testimonies and empirical findings supported the viability of the proposed approach. Especially, MAE of the rating prediction was 0.8011 with 60% training dataset size, while the error rate was equal to 0.7965 with 80% training dataset size. Moreover, MAE rates were 0.8781 and 0.9046 in new 50 and 100 customer cold-start scenarios, respectively. The proposed model outperformed other baseline models that independently employed the major properties of customers, products, or tags in the prediction process.

## 1. Introduction

Currently, information overload has become an issue because of the advancement of Internet technology and the influx of data from all domains. Numerous well-known websites and e-commerce platforms utilize a variety of practical and efficient recommender systems (RS) to address this issue, enhance their level of customer care, and attract and maintain regular customers. For instance, TikTok and Instagram social networks, Netflix movie recommendations, the AppStore and Play Market marketplace, YouTube online videos, etc. Thus, customers can obtain more relevant content due to the support of the recommendation algorithms in speeding up searches. Recommendation systems are created based on the collected data; therefore, their deployment and architecture are impacted by the diversity of data. Content-based filtering (CBF) and collaborative filtering (CF) [1,2,3] are two conventional methods for developing recommendation systems. When providing recommendations, CF-based approaches [4,5] exploit the products that customers have rated to anticipate unrated objects. These predictions are then automated by obtaining customer perceptions from the intended audience. All types of recommender system methods and approaches must prevent and handle [6] the cold-start problem and data sparsity, which are ongoing problems in the recommender system research field that can affect CF-based recommender systems. The rating matrices are unable to generate predictions due to data sparsity, which is brought about by less customer activity with products in a customer–product rating matrix. As a result, just 5 to 20 percent of matrices are contained with ratings. Additionally, the cold-start problem [7] occurs when there is inadequate knowledge of new customers and/or products to provide appropriate suggestions. CBF approaches create suggestions by examining customer–product interaction data that are available, which generally necessitates gathering explicit data [8,9]. For instance, content-based movie suggestions consider a film’s features that correspond to the customer’s previous preferences. Finding a link between movies and customers is crucial. On the other hand, researchers and developers attempt to design a recommendation system as a hybrid technique to improve recommendation accuracy [10,11,12] based on the combination of CF and CBF approaches to mitigate their individual limitations. In general, the aforementioned approaches to build an RS lead mostly to the cold-start problem, which has an impact on prediction accuracy when brought on by a lack of knowledge about new customers or objects. In addition, scalability and data sparsity issues arise in recommendation systems when the number of customers or products increases exponentially quickly; therefore, a recommendation technique should be quick and effective for big datasets. Therefore, clustering approaches aid in handling the sparsity problem more effectively and in reducing the processing time required for recommendation [13,14]. For example, several businesses, including Artsy, Netflix, and Pandora Internet Radio [15], have created unique clustering-based recommendation systems Art Genome Project, Micro-Genres of Movies, and Music Genome Project, respectively. In addition, many research works [16,17,18,19] have already been carried out on clustering and learning representative features of users in terms of similarity, which are important for modeling recommender system. Without regard to whether the length of the music list consumed is short or not, ref. [20] created a music clustering model to extract the interest points for a music recommendation system without having to predetermine the number of clusters. Bharti et al. [21] developed a model to deliver the best and fastest recommendations by maintaining and clustering current users and items of the system. Triyanna et al. [22] also proposed a recommendation model that integrates clustering technique and user behavior score-based similarity to reduce model computation complexity. To avoid the data sparsity problem, the research [23] presented a general framework to cluster users with respect to their tastes when the registers stored about the interactions between users and products are extremely scarce. Liu et al. [24] presented a clustering-based recommendation model that explores knowledge transfer and further aids the inferences about user interests.

Eventually, by leveraging tags and hierarchically organized hidden implicit features through early clustering and deep factorization, we attempted to solve the aforementioned problems in our study, thereby enhancing the performance of recommender systems as a whole. Generally, modern approaches for recommending videos mainly rely on ratings, textual data from the video such as labels (i.e., tags, reviews), or generating features from genre categories.

Specifically, by giving it to the prediction algorithm as a value, tag information, which consists of brief expression or words provided to movies by a customer who represents their affiliations or behavior, makes predictions easier. To enhance outcomes and address concerns with data sparsity and product cold start, engineers have already commented the advantages of providing recommendations through tags [25,26,27]. In contrast, hidden information that is organized hierarchically makes it easier to purposefully reveal information about specific products or customers, such as product categories on e-commerce websites (e.g., AliExpress, Coupang, and Wish) or genre categories of movies on popular services (e.g., IMDb and Netflix) [28].

**Figure 1:**
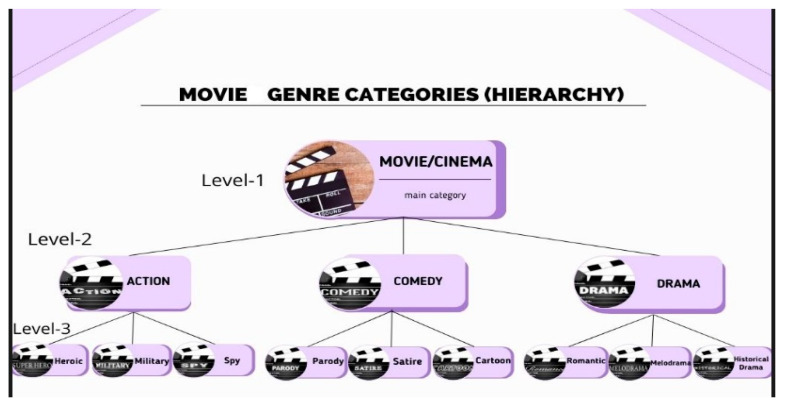
Movie categories.

Movies and customers of actual, useful recommendation systems may display certain hierarchical structures. A customer (female) in Figure 1 may, for instance, choose movies from the drama main category, or more precisely, she might choose movies from the romantic drama subcategory. Similar to that, the product (the Amazfit GTS 2 smart watch) may be classified as belonging to the subcategory “smart watches” under the general heading “electronics”. An object is categorized into the relevant lower-level categories or nodes in a progressive manner. They will probably receive comparable ratings owing to the likelihood that products at the same hierarchical level would have similar features. Customers of the same level in the hierarchy are equivalently more likely to have similar tastes, which makes it more probable that they would evaluate specific goods likewise [29]. For this reason, when it comes to large dataset, we took advantage of early clustering customer–product interactions and integrated simultaneously tag information and acquired hierarchically structured hidden information of products and customers for prediction process to mitigate the above-mentioned issues and improve overall RS performance. We investigated the hierarchical structures of customers and products for recommender systems in part due to the importance of hierarchically organized hidden information and their limited availability. For the purpose of developing mathematical model, the study was focused on obtaining products and customers’ hierarchical structures for generating recommendations. Additionally, it was researched how to combine customers’ tag annotation through mathematically obtained customers and products’ hierarchical structures to create a structured model that serves as the foundation for a recommender system. To the best of our knowledge, the customers and products’ hierarchical structured implicit features and tag information have never been used in conjunction based on early clustered customer rating matrix and deep factorization although extensive research has been done to show how two characteristics may be used individually in recommender systems.

In this article, a novel approach that employs clustering customers and deep factorization on customers and products procedure was proposed. Particularly, clustering technique is utilized to create customer groups with similar rating score history on products. After creating customer groups, the deep-factorization technique was applied to obtain hierarchically organized hidden implicit features of customers and products of one group, whereas the features used to predict ratings within group and tag information were combined synchronously as additional parameter to regularize the deep-factorization process. Clustering the customers as one group in early stage and deeply factorizing the customer–product interaction matrix in that group to produce hierarchical relationships of customers and products through regularizing the factorization process via tag information to predict ratings were the guiding theory behind the suggested approach.

Our primary contributions via the suggested approach were as follows:

- Create the smoothed dense rating matrix using early clustering;
- Obtain hierarchically structured implicit features of customers and products;
- Mathematically model the synchronous impact of hierarchically structured implicit features and tag information for recommendation;
- Regularize via the auxiliary parameter based on tag information;
- Minimize product cold start and data sparsity difficulties;
- Increase the overall performance of recommendation when a dataset is large.

The rest of this paper is formatted as follows. In Section 2, we discuss several studies on producing hierarchical features, accurate MF techniques, and clustering- and tag-based recommender systems. In Section 3 and Section 4, we go over the suggested approach in great depth and demonstrate its correctness through tests and comparisons against other methods. The results and scope of the future research are presented in Section 5. Finally, the referenced materials are cited, many of which are more contemporary works.

## 2. Literature Review

### 2.1. Clustering-Based Recommender Systems

Several strategies, largely based on clustering techniques, have been developed to avoid substantial job-specific feature engineering because of the dramatically increased size of the datasets. There are many research works and examples of pure advanced clustering methods [30,31,32,33] to cluster a dataset. Yunfan Li et al. [30] recommend a one-stage online clustering method that directly generates positive and negative instance pairs using data augmentation and afterwards projects the pairs in a feature space. The row and column spaces are used to perform the instance- and cluster-level contrastive learning, accordingly, by maximizing the similarities of positive pairings and reducing those of negative ones. Peng et al. [32] also developed a novel subspace deep clustering method to manage real data that do not have the linear subspace structure. Especially, in order to gradually map input data points into nonlinear latent spaces, the clustering methods learns a series of explicit transformations while maintaining the local and global subspace structure. Clustering techniques are applied as a first step to enhance the performance of recommender systems when customers suffer from information overload. In particular, based on customer evaluations generated by customers who are similar to target consumers, CF is a method that forecasts which products should be offered to target customers. Accordingly, we anticipate an improvement in forecasting precision owing to the early clustering of individuals with comparable characteristics. Therefore, there are many studies [34,35,36,37] related to the dependability of recommendations, variety and regularity, as well as the data sparsity on customer-preference matrices and shifts in customer personal tastes over period, which may help to solve recommendation systems. The authors of [37] presented a novel collaborative-filtering method that relies on clustering customer preferences to eliminate the effects of data scarcity. Customer groups were first created to differ between clients who had distinct tastes. Subsequently, based on the tastes of an active customer, a list of the nearest neighbors from the pertinent customer group (or groups) is then produced. The aim of [38] was to lower the cost of finding the closest neighbor using the k-means approach to cluster customers and potential projects. Moreover, the sparseness of the rating matrix of past customers and the cold start of new customers [7] restrict the practical usefulness of CF models. In other words, to address data heterogeneity and sparsity, [39] provided a combined filtering technique based on bi-clustering and information entropy.

It specifically uses bi-clustering to identify the dense modules of a rating matrix, followed by an information entropy metric to assess how similar a new customer is to the dense modules. As previously demonstrated, clustering can be used as a preventive measure before recommending products.

### 2.2. Recommender Systems Based on Tag and Hierarchically Organized Data

Recent research has taken advantage of tags and hierarchically organized features as additional characteristics to overcome concerns with data scarcity and cold starts in recommendation engines [25,40,41,42]. CF RS models are frequently used to predict ratings connected with customer’s previous experiences; however, they disregard expensive dormant features that avoid cold starts and sparse data problems, which in turn degrade performance. Because of this, supplemental features have been incorporated into the recommendation process by many studies [43,44,45]. A rich knowledge architecture, i.e., hierarchy with relationships, is frequently maintained through supplementary features. To increase recommendation accuracy and overcome the cold-start issue, Yang et al. [40] suggested an MF-based framework incorporating recursive regularization that examines the effects of hierarchically arranged features in customer–product interactions. In an attempt to discover more trustworthy neighbors, Lu et al. [42] created a framework that uses hierarchical relationships depending on the preferences of potential customers. The hierarchical product space rank (HIR) technique uses the product space’s inherent hierarchical structure to reduce data sparsity, which might impair the effectiveness of predictions [43]. Before providing recommendations, the majority of contemporary recommender systems comb through implicit and explicit features as relevant data, such as social information, photos, textual information, and ratings about products and customer qualities. Consequently, we can conclude that investigating tag data is crucial in recommendation systems because the data not only summarize the properties of products but also aid in determining customer preferences. As an illustration, to determine consumers’ preferred meal components and features [25], food suggestions are created using a model trained on a dataset of customer preferences obtained from tags and ratings provided in product forms. In their general solution, Karen et al. [27] suggested breaking down 3D correlations into three 2D correlations and modifying the CF algorithms to account for tags. In addition, Gilberto Borrego et al. [46] proposed a classification technique to recommend tags from topics in chat/message using NLP methods. Moreover, the research in [47] provided a semantic tagging strategy that makes use of Wikipedia’s knowledge to methodically identify content for social software engineering while also semantically grounding the tagging process. Despite the availability of advanced clustering methods [30,31,32,33], we aimed to show contribution of clustering technique with basic k-means algorithm to improve additionally the effectiveness of hierarchically organized hidden implicit features of customers and products in building a recommendation model.

Therefore, our proposed methodology is based on the early clustering of customer–product interactions and simultaneously integrating tag and hierarchically structured information into the rating-prediction process. In summary, existing MF models that use hierarchical and tag information individually deliver satisfactory results despite their complexity. However, to the best of our knowledge, there is no available advantageous study that seamlessly incorporates hierarchical and tag information simultaneously by early clustering customer–product interactions to improve the overall performance of the proposed recommender model. In summation, considering their complexity, current MF models that utilize tag information and hierarchically structured dormant implicit features separately produce good results. To the best of our knowledge, no useful study has yet been published that successfully combines the two data by early-clustering customer–product interactions to enhance the overall performance of the suggested recommender model.

## 3. The Proposed Approach

**Figure 2:**
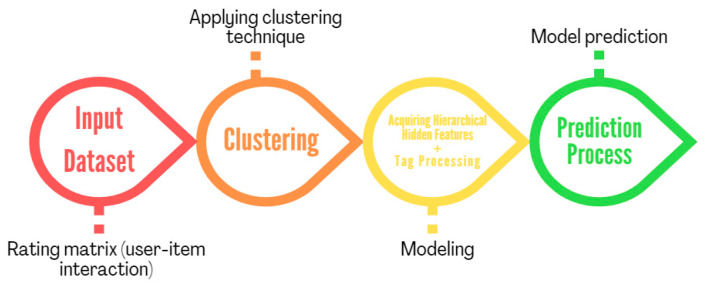
Modeling process.

This section illustrates our proposed methodology that clusters early customer–product interactions and predicts rating scores by acquiring hierarchical structured hidden features of products and customers simultaneously with a mathematically modeled combination of customers tag annotation. In particular, a foundational model that serves as the foundation for generating dormant features is detailed after the clustering approach employed in this model is introduced. The specifics of the model’s elements that mathematically represent the hidden, hierarchically organized dormant characteristics of products and consumers while also integrating tag data to produce an optimization issue are then discussed. Finally, a productive algorithm is provided for addressing this problem. The Figure 2 shows steps of the modeling process to reach the productive algorithm. 

The details of each component of the modeling process are provided in the following section.

### 3.1. Early Clustering

**Figure 3:**
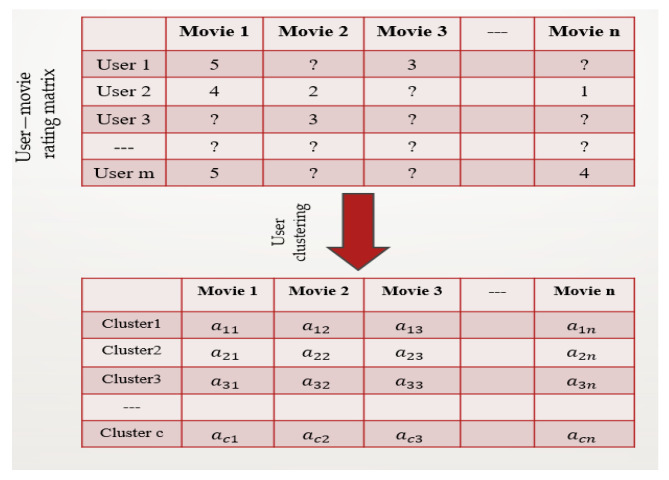
Customer clustering.

The time-consuming adjacent collaborative-filtering inquiry of the prospective customers in the whole customer domain results in the incapacity to guarantee the real-time need of recommender systems when customers and goods in e-commerce websites increasingly rise. Additionally, when the customer database’s record count increases, it loses quality owing to its poor design. The main factor contributing to the low quality was the sparseness of the original dataset. This research offers a customized recommendation technique that uses an early customer-clustering method to address the issues of scalability and sparsity in building recommendation systems. In this study, we concentrate on grouping comparable customers using k-means clustering as a first step, and then, we further enhance the efficacy of recommendations by gathering the hidden attributes of customers and things. Customers are grouped into clusters based on a customer–product rating matrix. The closest neighbors of the target customer may be identified and used to smooth the prediction as needed based on the similarity of the target customer and cluster centers. Customer-clustering techniques determine groups of customers who seem to have common ratings. Predictions for a target customer can be generated after the clusters have been formed by averaging the feedback from other customers in that cluster. Each customer is portrayed using certain clustering approaches as having varying degrees of membership in various clusters. Next, the weighted average of the predictions for each cluster is calculated. However, the performance can be quite good once customer clustering is finished, as the size of the group that has to be evaluated is significantly lower [48]. The concept employs a customer-clustering algorithm to partition customers of the collaborative-filtering system into neighborhoods, as shown in Figure 3. Depending on the similarity criterion, the clustering algorithm may produce divisions of a specific size or specified number of partitions of variable sizes.

A detailed Algorithm 1 for the early customer-clustering technique is presented as follows:
**Algorithm 1:** The early customer-clustering technique**Input:***User–item rating matrix, clustering number k* **Output:***The smoothed dense user–item matrix* **Start:** *Select user set* $u=\left\{u_{1},\;u_{2},u_{3}\;\ldots ,u_{m}\right\}$;  *Select item set i* $=\left\{i_{1},\;i_{2},i_{3}\;\ldots ,i_{n}\right\}$;  *Select the top k rating users as the clustering* $cu=\left\{cu_{1},\;cu_{2},cu_{3}\;\ldots ,cu_{m}\right\}$;  *The clustering center is null as* c $=\left\{c_{1},\;c_{2},c_{3}\;\ldots ,c_{k}\right\}$; *do*   for each user $u_{i}\in u$
    for each cluster center c$u_{i}\in cu$       calculate the similarity ($u_{i}$, c$u_{i}$);     end for     *sim*($u_{i}$, c$u_{m})\;=\;\max\left\{\mathrm{sim}(u_{i},\;cu_{1}),\mathrm{sim}(u_{i},\;cu_{2})\;\ldots ,\mathrm{sim}(u_{i},\;cu_{k})\right\}$;     $c_{m}=c_{m}\cup u_{i}$    *end for*    *for each cluster* $c_{i}\in c$      *for each user* $u_{j}\in u$      c$u_{i}=average\left(c_{i},u_{j}\right)$;      *end for*    *end for*   *while* (c is not change) **End**

Data sparsity is one of the difficulties associated with RS. In the customer–product rating dataset, we explicitly utilized customer clusters to which we applied our prediction technique for each individual cluster. By calculating the customer-clustering algorithm, we obtained dense customers who interacted with specific products. Therefore, the original sparse customer–product rating matrix then became a dense customer–product matrix in each cluster.

### 3.2. Founding Model

By grouping comparable customers using a customer-clustering algorithm, we obtained several customer clusters. Then, we applied our key idea to obtain hierarchically structured hidden features of customers in each cluster and related products. The core principle is a basic weighted nonnegative matrix factorization (WNMF) model, which is efficient and simple to use in recommender systems with huge and sparse datasets. Two nonnegative matrices P and Q with sizes n×r and r×m were created via the WNMF, which factorizes deeply clustered customer–product rating matrix.

(1) $$R^{\prime }\approx PQ=\left[\begin{array}{c} p_{1}\\ p_{2}\\ \ldots \\ \ldots \\ p_{n} \end{array}\right]\left[q_{1}q_{2}\ldots .,q_{m}\right]$$

The rating score assigned by $p_{i}$ to $q_{j}$ is then derived as $R^{\prime }\left(i,j\right)=P\left(i,:\right)Q\left(:,j\right).$ P and Q were estimated by resolving the following optimization issue:

(2) $$\underset{P,Q}{\underbrace{\min}}{\left\|W⊙(R-PQ)\right\|}_{F}^{2}+\lambda ({\left\|P\right\|}_{F}^{2}+{\left\|Q\right\|}_{F}^{2})$$

where W is the hyperparameter that balances the aid of $R^{\prime }\left(i,j\right)$ in the learning process such that W(i,j)=1 for $R^{\prime }\left(i,j\right)>0$; else, W(i,j)=0. ⊙ is the Hadamard element-wise multiplication operator, λ is the regularization parameter applied to alleviate the overfitting and intricacy under learning, and ${\left\|P\right\|}_{F}^{2}$ and ${\left\|Q\right\|}_{F}^{2}$ are the Frobenius norms of the respective matrices [27].

### 3.3. Generating the Implicit Dormant Features

Customers and products have hidden and hierarchically organized implicit features. Figure 1 depicts a hierarchical structure for organizing film genres as one example. If we illustrate films as categorized, it is highly probable that films in detailed genres have more in common with one another than films in subgenres. Therefore, a film within the same specific genre as one with a high customer rating score must be appropriate to suggest. The overall performance of recommendations could be even more strengthened by synchronously acquiring the supplementary features included in hierarchical customer and product structures. The fundamental WNMF model is deeply factorized in order to obtain the hierarchically organized hidden implicit features of customers and products. Moreover, they can be mathematically modelled for the rating-prediction process based on the following theory.

**Figure 4:**
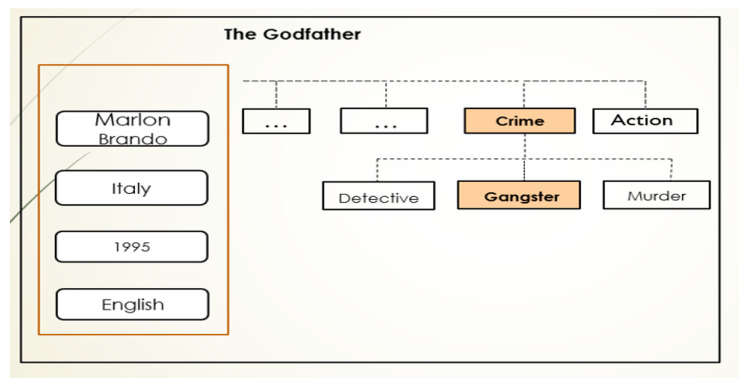
Movie genre categories and other flat features.

The theory here is that:

- Products with similar features within the same hierarchical level are more likely to be given identical ratings.
- Customers within the same hierarchy level are more likely to have similar tastes, which makes it probable that they would score particular products identically.
- Thus, in this subsection, the way of generating hierarchically structured hidden implicit features of customers and products is represented with the WNMF. Finding useful information from the characteristics of highly linked customers and products in their interaction, which serves as the foundation for the prediction process, is one of the biggest problems of recommendation systems. However, these characteristics are commonly depicted in a hierarchy, i.e., a multilevel structure, as a nested tree of nodes (for instance, film genres or customer profession). Film genres and product categories on e-commerce websites are straightforward illustrations of a hierarchical structure. For instance, the film *The Godfather* (a product) may be categorized by moving through the nodes of the hierarchical tree as shown in Figure 4: main category → subcategory, which appears as Crime → Gangster.

**Figure 5:**
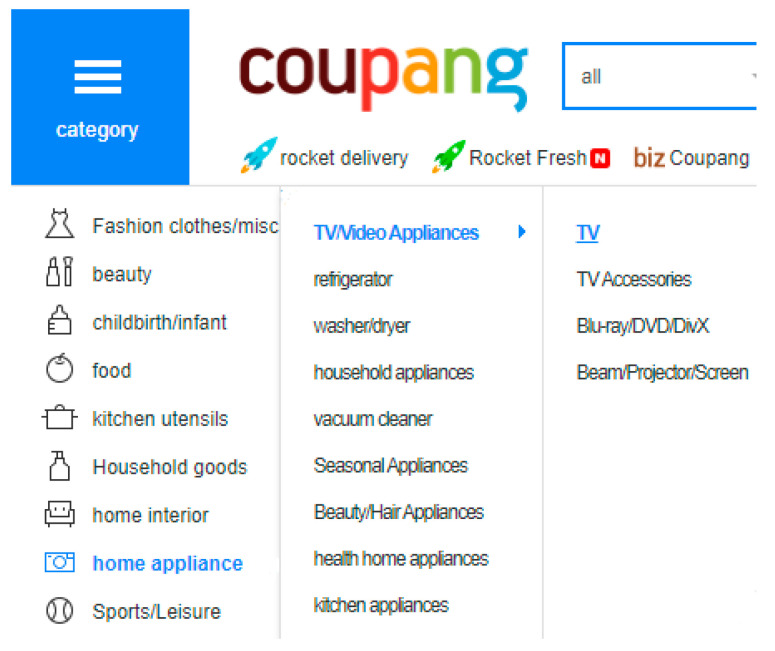
Coupang product categories.

In a similar manner, LG OLED 4K TV (a product) can be classified in a hierarchical structure as Home appliance > TV/Video appliances > TV (primary category → subcategory → explicit subcategory), as shown in Figure 5.

Customer preferences follow a similar pattern. For example, a customer who prefers to score crime films may like the gangster in subgenre above others, and customers who regularly score products that have similar qualities when browsing a product catalog may be expressing coincidental preferences. In order to extract implicit hidden hierarchical features of customers and customers and then anticipate rating scores, the WNMF primary model described in Section 3.2 was utilized. The customer–product rating matrix in each cluster was broken down into two nonnegative matrices, **P** and **Q**, which indicate customer preferences and product features, accordingly, and are stated as flat features. **P** and **Q** are nonnegative; thus, we factored them using a nonnegative matrix to understand the related hierarchically organized features, which allowed us predict the rating scores provided by Formula (1). In order to identify the latent projections of n customers and m products in an r-dimensional latent category, P and Q were retrieved so that $P\in {\mathbb{R}}^{n\times r}$ and $Q\in {\mathbb{R}}^{r\times m}$ were created (space). Because P and Q are nonnegative, they could be additionally factorized to mimic the hierarchical structure. Consequently, in a certain implementation, P is factorized into two matrices, $P_{1}\in {\mathbb{R}}^{n\times n_{1}}$ and ${\tilde{P}}_{2}\in {\mathbb{R}}^{n_{1}\times r}$, as follows:

(3) $$P\approx P_{1}{\tilde{P}}_{2}$$

where n is the quantity of customers, r is the quantity of latent categories (space) in the main (first) hierarchically organized layer, and $n_{1}$ is the quantity of subcategories in the next (second) hierarchically organized layer. Thus, $P_{1}\in {\mathbb{R}}^{n\times n_{1}}$ depicts the association between n customers and $n_{1}$ subcategories. ${\tilde{P}}_{2}$ stands for the second hierarchically organized layer of the customers’ hierarchical structure, which was determined by relating the quantity of latent categories (space) in the main (first) hierarchically organized layer to the quantity of latent subcategories in the hierarchically organized layer. Formula (4) provides customer’s third hierarchically organized layer, and then, ${\tilde{P}}_{2}$ is additionally factorized as $P_{2}\in {\mathbb{R}}^{n_{1}\times n_{2}}$ and ${\tilde{P}}_{3}\in {\mathbb{R}}^{n_{2}\times r}$:

(4) $$P\approx P_{1}P_{2}{\tilde{P}}_{3}$$

where $n_{2}$ is the quantity of subcategories in the third hierarchically organized layer. Thus, deep factorization on **P** is used to determine the customer’s x-th hierarchically organized layer. $P_{x}$ is carried out by factorizing ${\tilde{P}}_{x-1}$, the latent category relationship matrix of the (x−1)th layer of the hierarchical structure, into nonnegative matrices as follows:

(5) $$P\approx P_{1}P_{2}\ldots \ldots \ldots \ldots P_{x-1}P_{x}$$

where $P_{i\;}\geq 0$ for i∈ {1,2,…,x}, $P_{1}$ is an $n\times n_{1}$ matrix such that $P_{i}$ is an $n_{i-1}\times n_{i}$ matrix, and $P_{x}$ is $n_{x-1}\times r$ matrix.

**Figure 6:**
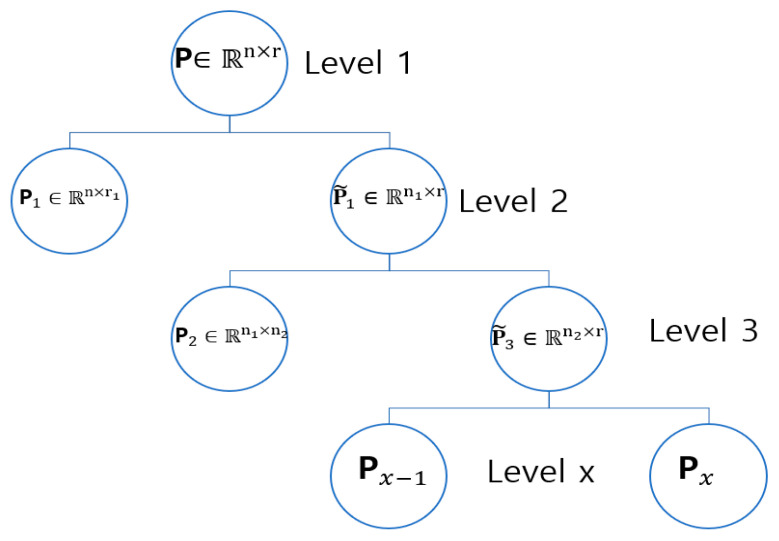
Generating customer’s implicit dormant features.

For Q, the aforementioned factorization procedure (Figure 6) was replicated to acquire hierarchically structured implicit features of the products. For that, the association of m products with r-dimensional latent categories (space) is depicted as $Q\in {\mathbb{R}}^{r\times m}$, which is additionally factorized into $Q_{1}\in {\mathbb{R}}^{m_{1}\times m}$ and ${\tilde{Q}}_{2}\in {\mathbb{R}}^{r\times m_{1}}$ to characterize products’ the hierarchically organized layer in the hierarchy as follows:

(6) $$Q\approx {\tilde{Q}}_{2}Q_{1}$$

where $m_{1}$ is the quantity of sub-categories in the second hierarchically organized layer, and $Q_{1}\in {\mathbb{R}}^{m_{1}\times m}$ is the association of m products to the $m_{1}$ latent subcategories. The latent category association of the nonnegative matrix ${\tilde{Q}}_{2}\in {\mathbb{R}}^{r\times m_{1}}$ of the second hierarchically organized layer is defined as the affiliation between r-dimensional latent categories (space) in the first hierarchically organized layer and $m_{1}$ latent subcategories in the second hierarchically organized layer. Formula (7) provides the third hierarchically organized layer of products, where ${\tilde{Q}}_{2}$ is also factorized as $Q_{2}\in {\mathbb{R}}^{m_{2}\times m_{1}}$ and ${\tilde{Q}}_{3}\in {\mathbb{R}}^{r\times m_{2}}$, where $m_{2}$ is the number of subcategories in the third hierarchically organized layer:

(7) $$Q\approx {\tilde{Q}}_{3}Q_{2}Q_{1}$$

**Figure 7:**
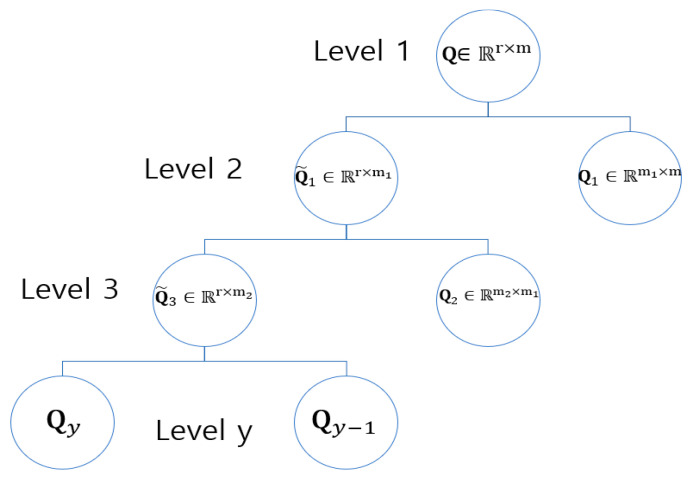
Generating product’s implicit dormant features.

As shown in Figure 7, carrying out the deep-factorization process with Q assures the products’ y-th hierarchically organized layer, $Q_{y}$ which is accomplished by factorizing ${\tilde{Q}}_{y-1}$, in the (y−1)th layer of the hierarchy, as follows:

(8) $$Q\approx \;Q_{y}Q_{y-1}\ldots Q_{2}Q_{1}$$

where $\;Q_{j\;}\geq 0$ for j∈ {1,2,…,y}, $Q_{1}$ is an $m_{1}\times m$ matrix such that $Q_{j}$ is an $m_{j}\times m_{j-1}$ matrix, and $Q_{y}$ is an $r\times m_{y-1}$ matrix.

Conclusively, to create a systematic model that depicts the products and customers’ hierarchically organized layers, the following optimization issue must be solved:

(9) $$\underset{P_{1},\ldots P_{x},Q_{1}\ldots .Q_{y}}{\underbrace{\min}}{\left\|W⊙(R-P_{1}\ldots P_{x}Q_{y}\ldots Q_{1})\right\|}_{F}^{2}+\lambda (\overset{x}{\underset{i=1}{\sum }}{\left\|P_{i}\right\|}_{F}^{2}+\overset{y}{\underset{j=1}{\sum }}{\left\|Q_{j}\right\|}_{F}^{2})$$

where $P_{i\;}\geq 0$ for *i* ∈ {1,2,…,x}, and $Q_{j\;}\geq$ 0 for *j* ∈ {1,2,…,y}.

**Figure 8:**
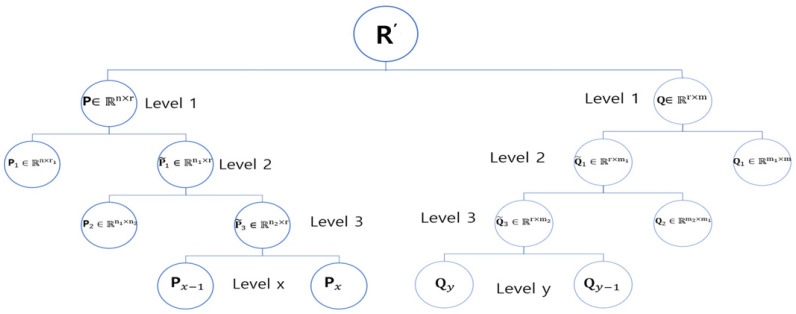
The design of generating products and customers’ implicit dormant features.

Figure 8 illustrates the rating-prediction approach that generates the products and customers’ hierarchically organized layers.

### 3.4. Integrating Customers’ Tag Annotation

While customer ratings are considered as the main data source for a rating-prediction process, the customers’ characteristics or products’ properties are not considered in most research works. In this case, tags offer valuable auxiliary information for recommender systems because they represent customer preferences or product characteristics. In addition, tag information plays a crucial role in recommendation systems; obviously, it is natural customer-generated resource text data that express customers’ interests in various ways towards products. Customers who post similar tags are likely to have similar interests; therefore, they are likely to give similar ratings to products. The auxiliary information provided by tags leads to the advancement of recommendation systems to the next level. Tag information is a word or a short phrase for products given by customers. Thus, customers’ preferences for products may be indirectly expressed by the tags, and this tag information could offer valuable information for the movie prediction process. Therefore, in order to infer a correlation between the supplemental information requested from WNMF and tag constant repetition in products [5], tag information was specifically integrated into our suggested technique. For instance, a customer’s “organized crime” tag applied to the movie *The Godfather* (product) may also be applied to other items with comparable features, which is represented in the degree of repetition.

**Figure 9:**
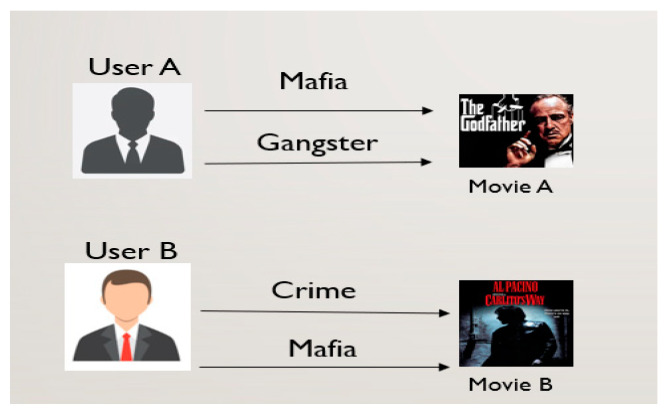
Tag annotation.

**Figure 10:**
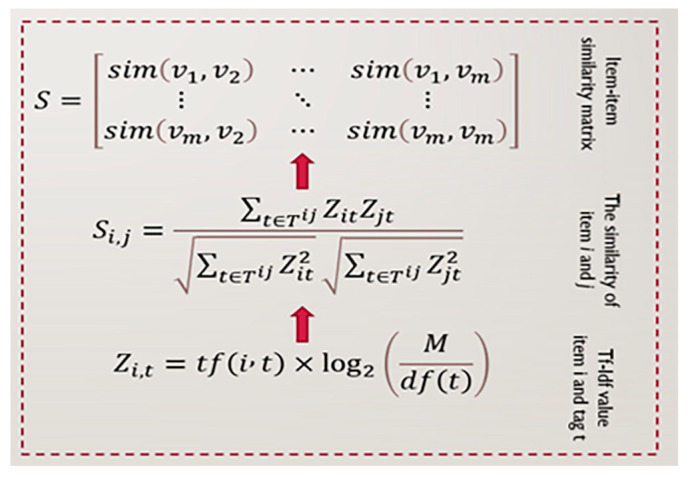
Tag processing part.

As illustrated in Figure 9, customer A often uses tags Mafia and Gangster, whereas customer B uses tags Crime and Mafia; hence, both customers may like movies A and B. Here, the intersection of the tagging history between customers is the Mafia tag. Hence, they had a similar tagging history. Therefore, a similar tagging history may indicate a similar customer’s personal interest in products and/or similarities between products. Additionally, tags can be seen as product descriptions, which may help define a product’s character or nature. The purpose of using tag information is to find similarities between products based on the tag information as illustrated in Figure 10 and then use product similarity as an additional parameter to organize the factorization process. The idea behind incorporating tag information is to use product similarities based on tag information to regularize the factorization process of the proposed prediction model. Therefore, the matrix factorization process of the weighted nonnegative matrix factorization model is regularized based on tag information. For clarity, we formed two product-specific latent feature vectors that are as similar as possible if the two products have similar tag information.

Thus, in order to finish our rating-prediction approach, tag information is utilized to regularize the deep-factorization process of a fundamental WNMF model. In essence, we want to create two similar-natured, product-specific latent feature vectors from our fundamental WMNF model’s factorization process. These vectors would comprise products with comparable tag information. Each tag information matrix **T** with components $T_{it}$ for product i and tag t is a tf∗idf value [49].

(10) $$T_{\mathrm{it}}=\mathrm{tf}\left(i,t\right)*\log_{2}\left(\frac{m}{\mathrm{df}\left(t\right)}\right)$$

where tf(i,t) is the normalized frequency of t occurring in i, df(t) is the quantity of products containing t, and m is the total quantity of products. Thus, the similarity between products i and j is estimated using the cosine similarity formula given as follows:

(11) $$S_{i,j}=\frac{\sum_{t\in T^{ij}}T_{it}T_{jt}}{\sqrt{\sum_{t\in T^{ij}}T_{it}^{2}}\sqrt{\sum_{t\in T^{ij}}T_{jt}^{2}}}$$

where $T^{ij}$ is the index of tags occurring in both products i and j. The two product-specific latent feature vectors that are most similar are then obtained by affixing a product similarity regularization criterion function to the WNMF model as follows:

(12) $$\begin{array}{c} \frac{\beta }{2}\sum_{i=1}^{N}\sum_{j=1}^{N}S_{i,j}{\left\|q_{i}-q_{j}\right\|}_{F}^{2}=\frac{\beta }{2}\sum_{i=1}^{N}\sum_{j=1}^{N}\left[S_{i,j}\;\sum_{\;r\prime \;=1}^{r}{\left(q_{\;r\prime \;i}^{\;}-q_{\;r\prime \;j}^{\;}\right)}^{2}\right]\\ =\frac{\beta }{2}\sum_{\;r\prime \;=\;1}^{r}Q_{r^{\prime *}}LQ_{r^{\prime *}}^{T}=\frac{\beta }{2}\mathrm{tr}\left(QLQ^{T}\right) \end{array}$$

where $S_{i,j}$ defines the similarity between i and j; $q_{1}q_{2}\ldots .,q_{m}$ are latent characteristic vectors that populate Q; r is the dimension of each product in the vector; i.e., $q_{\;r\prime \;i}^{\;}$ and $q_{\;r\prime \;j}^{\;}$ are the values of vector products i and j of the  r′ th dimension; L defines the Laplacian matrix given by L=D−S for a diagonal matrix D such that $D_{ij}=\sum_{j}S_{ij}.\;\mathrm{tr}\left(\cdot \right)$ is a trace of the matrix; β is an extra regularization parameter that controls the balance of the tag information [50].

Mixing Formulas (9) and (12) utilized for the rating-prediction process and the corresponding objective function is minimized optimally.

(13) $$\underset{P_{1},\ldots P_{x},Q_{1}\ldots .Q_{y}}{\underbrace{\mathrm{Min}}}{\left\|W⊙(R-P_{1}\ldots P_{x}Q_{y}\ldots Q_{1})\right\|}_{F}^{2}+\;\lambda \left(\sum_{i=1}^{x}{\left\|P_{i}\right\|}_{F}^{2}+\sum_{j=1}^{y}{\left\|Q_{j}\right\|}_{F}^{2}\right)+\frac{\beta }{2}\mathrm{tr}\left(QLQ^{T}\right)$$

where $P_{i}\geq 0$ for i∈ {1,2,..,x}, and $Q_{j\;}\geq 0$ for j∈ {1,2,..,y}.

### 3.5. Optimization

Any algorithm that determines the minimum or maximum of a function must first determine the best method for performing the rating procedure. Numerous studies have employed various optimization strategies and uncertainty simulation techniques in recent years to address optimization issues involving unknown factors. Because of the non-convexity of the objective function, optimization problems are inherently challenging tasks. The superiority of any approach that can be used in a recommendation system is also a result of the problem being solved. Thus, the switching operation [51] is utilized as our optimization technique. In particular, all variables are updated reciprocally in the abovementioned objective function, leading to the function becoming convex.

#### 3.5.1. Updating $P_{i}$

When $P_{i}$ is updated, terms distinct to $P_{i}$ are eliminated by fixing the remaining variables, and the last objective function is declared as

(14) $$\underset{P_{i}\geq 0}{\underbrace{\min}}{\left\|W⊙\left(R-A_{i}P_{i}H_{i}\right)\right\|}_{F}^{2}+\;\lambda \;{\left\|P_{i}\right\|}_{F}^{2}$$

where $A_{i}$ and $H_{i}$ for 1 ≤ i ≤ x are determined as:

(15) $$A_{i}=\{\begin{array}{ll} P_{1}P_{2}\ldots \ldots \ldots ..P_{x-1} & \mathrm{if}\;i\neq 1\\ \;I & \mathrm{if}\;i=1\; \end{array}$$

(16) $$H_{i}=\{\begin{array}{ll} P_{i+1}\ldots P_{x}Q_{y}\ldots Q_{1} & \mathrm{if}\;i\neq x\\ \;Q_{y}\ldots Q_{1} & \mathrm{if}\;i=x\; \end{array}$$

The Lagrangian function in Formula (14) is:

(17) $$L\left(P_{i}\right)={\left\|W⊙(R-A_{i}P_{i}H_{i})\right\|}_{F}^{2}+\lambda {\left\|P_{i}\right\|}_{F}^{2}-\mathrm{Tr}\left(M^{T}P_{i}\right)$$

where M indicates the Lagrangian multiplier. The derivative of $L\left(P_{i}\right)$ with respect to $P_{i}$ is then given by

(18) $$\frac{\partial L\left(P_{i}\right)}{\partial P_{i}}=2A_{i}^{T}\|W⊙\left(A_{i}P_{i}H_{i}-R\right)\|H_{i}^{T}+2\lambda P_{i}-M$$

By utilizing the Karush–Kuhn–Tucker complementary requirement [52,53] that is equal to 0, $M\left(s,\;t\right)P_{i}\left(s,t\right)=0$, we derive

(19) $$\left[A_{i}^{T}\left[W⊙\left(A_{i}P_{i}Q-R\right)\right]H_{i}^{T}+\;\lambda P_{i}\right]\left(s,\;t\right)P_{i}\left(s,t\right)=0$$

Lastly, the updated rule of $P_{i}$ is estimated utilizing

(20) $$P_{i}\left(s,t\right)\leftarrow P_{i}\left(s,t\right)\sqrt{\frac{\left[A_{i}^{T}\left(W⊙R\right)H_{i}^{T}\right]\left(s,\;t\right)}{\left[A_{i}^{T}\left(W⊙\left(A_{i}P_{i}H_{i}\right)\right)H_{i}^{T}+\;\lambda P_{i}\right]\left(s,\;t\right)}}$$

#### 3.5.2. Updating $Q_{i}$

Likewise, for $Q_{i\;}$, the distinct terms are initially eliminated by fixing the remaining variables, and the last objective function is declared as

(21) $$\underset{Q_{i}\geq 0}{\underbrace{\min}}{\left\|W⊙(R-B_{i}Q_{i}K_{i})\right\|}_{F}^{2}+\lambda {\left\|Q_{i}\right\|}_{F}^{2}+\frac{\beta }{2}\mathrm{tr}\left(QLQ^{T}\right)$$

where $B_{i}$ and $K_{i}$ for 1 ≤ i ≤ x are determined as:

(22) $$B_{i}=\{\begin{array}{ll} P_{1}\ldots P_{x}Q_{y}\ldots Q_{y+1} & \mathrm{if}\;i\neq y\\ \;P_{1}\ldots P_{x} & \mathrm{if}\;i=y\; \end{array}$$

(23) $$K_{i}=\{\begin{array}{ll} Q_{y-1}\ldots Q_{1} & \mathrm{if}\;i\neq 1\\ \;I & \mathrm{if}\;i=1\; \end{array}$$

We could then estimate the updated rule for $Q_{i\;}$ in the same way as $P_{i\;}$:

(24) $$Q_{i}\left(s,t\right)\leftarrow Q_{i}\left(s,t\right)\sqrt{\frac{\left[B_{i}^{T}\left(W⊙R\right)K_{i}^{T}+\frac{\beta }{2}\mathrm{tr}\left(QLQ^{T}\right)\right]\left(s,\;t\right)}{\left[B_{i}^{T}\left(W⊙\left(B_{i}Q_{i}K_{i}\right)\right)K_{i}^{T}+\;\lambda Q_{i}+\frac{\beta }{2}\mathrm{tr}\left(QLQ^{T}\right)\right]\left(s,\;t\right)}}$$

The approximation of the components in the suggested approach is expected to be revealed through optimization using the aforesaid updating strategies for $P_{i}$ and $Q_{i}$. In order to derive a preliminary estimation of the matrices $P_{i}$ and $Q_{j}$, every hierarchically organized layer is pretrained. The customer–product rating matrix in each cluster is factorized into ${\tilde{P}}_{i}\;{\tilde{Q}}_{i}$ by calculating Formula (2). ${\tilde{P}}_{i}$ and ${\tilde{Q}}_{i}$ are then additionally factorized into ${\tilde{P}}_{i}\approx P_{1}{\tilde{P}}_{2}$ and ${\tilde{Q}}_{i}\approx {\tilde{Q}}_{2}Q_{1}$, respectively. The deep-factorization process is maintained until the pth customer and qth product hierarchically organized layers are acquired. The fine-tuning is accomplished by updating $P_{i}\;\mathrm{and}\;Q_{i}$ utilizing Formulas (20) and (24) accordingly: The initial movement covers updating $Q_{i}$ in order and then $P_{i}$ in sequence. Lastly, the proposed prediction rating matrix is equal to $R^{\prime }=P_{1}\ldots P_{x}Q_{y}\ldots Q_{1}$**.**

### 3.6. Convergence Analysis

The suggested approach’s convergence was examined using the following methodology. The aide function in [54] was utilized to demonstrate the approach’s convergence.

**Definition** **1.** *The aide function [54] is determined as $G\left(h,h^{\prime }\right)$* *for F(h)* *if the following criteria are met.*

(25) $$G\left(h,h^{\prime }\right)\geq \;F\left(h\right),\;G\left(h,h\right)=F\left(h\right)\;$$

**Assumption** **1.** *If G [54] is an aide function for F, then F is nonincreasing under the update.*

(26) $$h^{\left(t+1\right)}=\arg\min G\left(h,h^{\left(t\right)}\right)\;$$

**Proof** **.** (27) $$F\left(h^{t+1}\right)\leq G\left(h^{\left(t+1\right)},h^{\left(t\right)}\right)\leq G\left(h^{\left(t\right)},h^{\left(t\right)}\right)\leq G\left(h^{\left(t\right)}\right)$$

□

**Assumption** **2.** *For any matrix $A\in {\mathbb{R}}_{+}^{n\times n}$, $B\in {\mathbb{R}}_{+}^{k\times k}$,$\;S\in {\mathbb{R}}_{+}^{k\times k}$, and $S^{\prime }\in {\mathbb{R}}_{+}^{k\times k}$, where **A** and **B** are symmetric [55,56], the following inequality holds:*

(28) $$\sum_{s=1}^{n}\sum_{t=1}^{k}\frac{\left(AS^{\prime }B\right)\left(s,t\right)S^{2}\left(s,t\right)}{S^{\prime }\left(s,t\right)}\geq Tr\left(S^{T}ASB\right)\;$$

By introducing quadratic terms and eliminating terms that are distracting to $P_{i}$, the objective function in Formula (14) may be expressed as follows:

(29) $$J\left(P_{i}\right)=\mathrm{Tr}\left(-2A_{i}^{T}\left(W⊙R\right)H_{i}^{T}P_{i}^{T}\right)+\mathrm{Tr}\left(A_{i}^{T}\left(W⊙\left(A_{i}^{T}P_{i}H_{i}\right)\right)H_{i}^{T}P_{i}^{T}\right)+\mathrm{Tr}\left(\lambda P_{i}P_{i}^{T}\right)$$

**Theorem** **1.** 
(30) $$\begin{array}{c} G\left(P,P^{\prime }\right)=-2\sum_{s,t}\left(A_{i}^{T}\left(W⊙R\right)H_{i}^{T}\right)\left(s,t\right)P_{i}\left(s,t\right)\left(1+\log\frac{P_{i}\left(s,t\right)}{P_{i}^{\prime }\left(s,t\right)}\right)\\ +\sum_{s,t}\frac{\left(A_{i}^{T}\left(W⊙\left(A_{i}^{T}P_{i}H_{i}\right)\right)H_{i}^{T}\right)\left(s,t\right)P_{i}^{2}\left(s,t\right)}{P_{i}^{\prime }\left(s,t\right)}+Tr\left(\lambda P_{i}P_{i}^{T}\right) \end{array}$$

The above function is an aide function for $J\left(P_{i}\right)$. Moreover, it is a convex function in $\left(P_{i}\right),$ and its global minimum is

(31) $$P_{i}\left(s,t\right)\leftarrow P_{i}\left(s,t\right)\sqrt{\frac{\left[A_{i}^{T}\left(W⊙R\right)H_{i}^{T}\right]\left(s,t\right)}{\left[A_{i}^{T}\left(W⊙\left(A_{i}P_{i}H_{i}\right)\right)H_{i}^{T}+\lambda P_{i}\right]\left(s,t\right)}}$$

**Proof.**  The confirmation is identical to that of [55]; thus, the details are skipped.□

**Theorem** **2.** *Updating $P_{i}$* *using Formula (20) monotonically decreases the value of the objective in Formula (13).*

**Proof.**  With Assumption 1 and Theorem 1, we have:

(32) $$J\left(P_{i}^{\left(0\right)}\right)=G\left(P_{i}^{\left(0\right)},P_{i}^{\left(0\right)}\right)\geq G\left(P_{i}^{\left(1\right)},P_{i}^{\left(0\right)}\right)\geq J\left(P_{i}^{\left(1\right)}\right)$$

□

Particularly, $J\left(P_{i}\right)$ reduces monotonically. Analogously, the update rule for $Q_{i}$ monotonically reduces the value of the objective in Formula (13). We can demonstrate that the optimization technique of the suggested approach converges since the value of the objective in Formula (13) is at best edged by “0”.

## 4. Model Evaluation

### 4.1. Data Preparation

**Figure 11:**
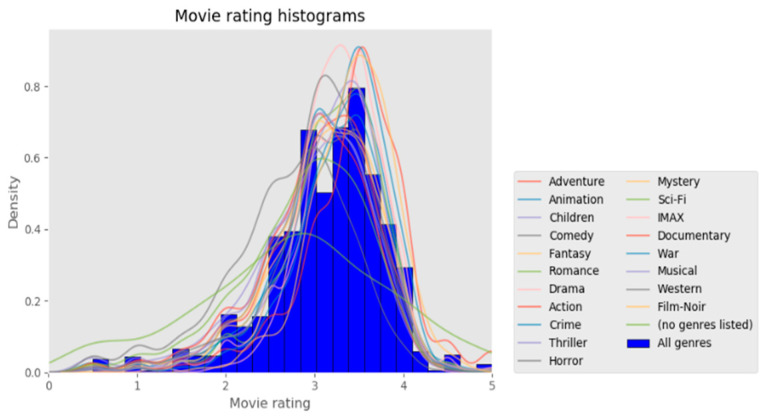
Ratings distribution in MovieLens 20M.

The design of recommendation systems is based on the kind of information acquired, and therefore, the variety of information affects how they are developed and are organized. Finding accurate and insightful data is thus the main goal of developing a recommender system. For recommendation systems, numerous datasets are obtainable, each of which includes different kinds of data. In this study, we developed our suggested recommendation algorithm and assessed its efficacy using the MovieLens 20M dataset. Customers were selected randomly for the dataset. At least 20 films were rated by each of the selected customers. The MovieLens online movie recommendation service’s 138,493 users assigned 20,000,263 ratings (Figure 11) and 465,564 tags to 27,278 films in the MovieLens 20M dataset. Each client gave a movie a rating between 1 and 5, with 5 being the greatest and 1 being the worst. For inclusion, the customers were chosen at random. At least 20 movies were rated by each chosen customer. MAE (mean absolute error) and precision/recall were chosen as measurement metrics to evaluate the proposed approach’s prediction accuracy, top N performance, and user cold-start problem.

**Figure 12:**
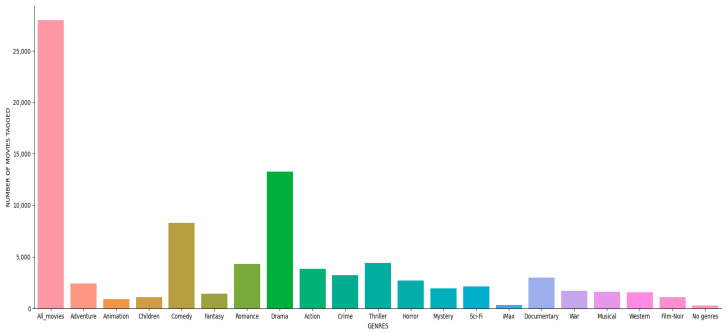
Number of tagged movies in each genre in MovieLens 20M.

In the dataset, all film genres had a similar tendency (right-skewed log-normal distribution), with the possible exception of horror films, which had a minor leftward skew (poorer ratings). Figure 12 shows the distribution of the tagged movies by genre.

### 4.2. Model Parameters

In this paper, the proposed approach tries to learn the customer–product interaction with the main optimal parameters in the given Table 1. The model was learned and showed the best results with the following parameters. The number of users and movies in the 1st hierarchical level ranged from {50, 100, 150, 200, 250} and {100, 200, 300, 400, 500}, accordingly. The value of the parameter r (number of movie genres) was “20”. The values of levels x and y in the hierarchy were similar, which effected to the model performance with optimal value “2”. To balance the deep-factorization procedure, the tag-based auxiliary regularization parameter revealed its strength on the performance, reaching a lowest error between 0.9 and 2.3. Thus, the optimal degree of auxiliary regularization parameter was taken with “1.7” value.

### 4.3. Experimental Conclusions

The proposed approach was evaluated via the best recommendation system indicators, including the rating-prediction error, extent of mitigating the user cold-start problem, and top-N performance results. It is important to note that the all experiments conducted to confirm and compare its advantage over other chosen baseline recommendation models, where the outcomes are illustrated in Table 2, Table 3 and Table 4.

#### 4.3.1. The Model Prediction Error

1. MF—matrix factorization: modeled by Koren et al. [5]; to reduce the difference between the anticipated and actual ratings, this approach factorizes a rating matrix and then acquires the resulting product and customer latent feature vectors.
2. WNMF—weighted nonnegative matrix factorization: the method is the basis of the suggested approach as a founding method to generate implicit dormant features. The WNMF tries to factorize a weighted rating matrix into two nonnegative matrices to reduce the difference between the anticipated and actual ratings.
3. F-ALS—fast alternating least squares matrix factorization: in order to decrease run-time and increase model efficiency than simple MF, the approach aims to create a model with more latent components to learn rating matrix.
4. BOW-TRSDL: the method attempts to develop product and customer’s profiles with benefits of bag-of-words (BOW) as the first step. Afterwards, DNN (deep neural networks) is utilized to retrieve the customers and products’ latent features, and then, these features are used to predict ratings.

#### 4.3.2. User Cold-Start Decision

The cold-start dilemma explains situations where a recommendation system is unable to provide pertinent suggestions since there are no ratings yet. This problem could make collaborative-filtering RS less effective. Cold-start issues are especially common with collaborative-filtering algorithms in particular. For this reason, the suggested solution can reduce the problem of user cold-start if user tag annotation and hierarchically organized implicit features are both available for usage. Furthermore, early-clustering customers provide a user group who have more similar interests on specific products. In addition to containing a description of the products, tags also provide user sentiment. Therefore, new customers without any preferences for any products can receive recommendations from it. The characteristic “profession” is uniquely hierarchically organized, and there is a connection between the layers. Customers in the same hierarchical tier are expected to have comparable traits, and consequently, it is likely that they will rate products similarly. As a result, the connections between consumers at various levels of the hierarchical structure produce rating forecasts and perhaps serve as additional implicit characteristics. A new user of the system is specifically positioned in a database based on their occupation. New customer locations in the industry create new connections between returning customers and a new client, and these connections will provide more data to forecast ratings for new clients. This was put to the test by treating 50 and 100 randomly chosen customers of the 80% training dataset as labeled new customers by ignoring their ratings. The proposed approach, which makes early-grouping customers and generates customers and products’ hierarchically organized implicit dormant features by integrating customer’s tag annotation to the prediction procedure in the state of the customer cold-start problem, exceeded competitive models and confirmed its comparative outcomes (Table 3) with the carried-out tests. It is noticeable that the proposed approach surpassed its counterparts and succeeded in alleviating the cold-start issue in both 100 and 200 new customer cold-start situations.

#### 4.3.3. Top-N Performance

The proposed approach also succeeded at the top N recommendations test in addition to delivering outstanding MAE scores on rating prediction. The films that perfectly matched the customer’s interests were found through experiments on the top N recommendations test. These films were determined by having hierarchically organized hidden implicit dormant features and customers’ tag annotation to the films. The higher-rated films were listed as top 10 recommendations for each customer, utilizing 80% training dataset to assess the proposed approach’s top N performance. The most popular 20M dataset was utilized to compare the proposed approach to other benchmark cutting-edge approaches, as shown in Table 4. In the comparison scenario, the proposed approach’s top 10 performance was satisfactory and succeeded due to early clustering the sparse large dataset and applying the dormant features into recommendations. Specifically, the top 10 performance was superior among the methods with a precision of 0.3405 and a recall of 0.2371 by requiring expensive operations for the initialization and fine-tuning processes. On the other hand, WNMF had the lowest performance and obtained 0.2694 precision and 0.1375 recall values, whereas these values for F-ALS were 0.2984 and 0.1851, accordingly. Moreover, MF achieved 0.3247 precision and 0.2053 recall top 10 performance results. However, the BOW-TRSDL model had close results in both precision and recall to the proposed model, with 0.3402 precision and 0.2113 recall results. Based on the experiment, it could be confirmed that the proposed method still worked successfully, and its superiority was clearly verified.

## 5. Conclusions and Future Scope

Although the evolution of customized recommender systems has progressed to a significant degree, there are still outstanding difficulties in the recommendation system field that need to be resolved, including data sparsity, cold starts, and enhancing recommender systems performance. This research suggests a unique rating-prediction approach that uses dormant implicit information and is based on deep factorization and early clustering. This model addresses the prediction performance, customer cold-start, and data sparsity difficulties in developing an efficient recommender system. First, a thorough examination of the underlying concepts, advantages, categories, and current issues of recommendation systems was conducted. Subsequently, a variety of relevant studies on clustering techniques, deep-factorization models, tag data, and hierarchically organized features were reviewed and assessed to lay the groundwork for the research project under consideration.

Employing implicit customer and product information plays a key role in improving the RS standard for online companies. In particular, gathering hierarchically organized hidden characteristics of persons and products enables one to overcome RS constraints and has been demonstrated to be essential in several research studies. Further, intentionally obtained tag data provide value to RS’s hierarchically organized hidden features and aid in improving the prediction model’s learning process by capturing the essence of customer–product interactions. The proposed approach in this study attempts to acquire hierarchically structured hidden implicit dormant features of customers and products and combine them via customers’ tag annotations. This regularizes the matrix factorization process of a fundamental weighted nonnegative matrix factorization (WNMF) model. The concept behind the proposed method is to regularize the process by utilizing customers’ tag annotations as a supplementary parameter to extract hidden hierarchical aspects of customer preferences and product attributes that indicate a deep link between them. The experimental results demonstrated a significant improvement in the rating-prediction process and product cold-start problem mitigation over previous MF systems when hierarchical features and tag information were combined.

Only in the case of products forming tag information with the hierarchical information of customers and products was the entire process of our suggested model for completion of rating predictions. Owing to their non-negativity, the customer preference and product characteristic matrices underwent deep factorization to produce hidden-level hierarchically organized features. To complete our prediction model, a straightforward matrix factorization process for the WNMF model was regularized using tag information. During the experimental testimony phase, we found that the efficiency of the proposed model initially improved and subsequently degraded when the values of the dimensions varied.

The advantage gained through this integration is that the designed model overcomes the data sparsity and user cold-start problems by early clustering customers based on the customer rating matrix. Furthermore, hierarchically structured hidden features are obtained and integrated with tag information for the prediction process in each customer cluster. Additionally, the experiments on the proposed model were conducted on the established MovieLens 20M dataset and proved that the proposed model is effective in improving the accuracy of top N recommendations with resistance to rating sparsity and cold-start problems when compared to the state-of-the-art CF-based recommendation models. Especially, the MAE of the rating prediction was 0.8011 with 60% training dataset size, while the error rate was equal to 0.7965 with 80% training dataset size. Moreover, MAE rates were 0.8781 and 0.9046 in new 50 and 100 customer cold-start scenarios, respectively. In terms of top 10 recommendations, precision and recall were 0.3405 and 0.2371. This indicates that our proposed model is effective in improving the accuracy of rating predictions and top N performance and alleviating the customer cold-start problem.

In reality, the adaptability and variety of the underlying idea, which make contributions to a number of topics, are seen as its greatest strengths. The world of recommender systems is not necessarily the only one to which these contributions apply. It is now obvious how crucial and successful recommender systems are for modern Internet enterprises, and the suggested algorithm has room for development.

The following are some directions for further research:

- To design a recommender system that is understandable and comprehensible using implicit hidden characteristics;
- To use metaheuristic techniques to enhance performance metrics [57];
- To handle the “grey sheep” issue, which occurs when a customer cannot be matched with any other customer group, and the system is unable to produce helpful recommendations [58];
- To provide dynamic predictions with the least amount of complexity;
- To develop an emotion-based movie recommendation model [59,60];
- To integrate other advanced clustering methods such as twin contrastive learning for online clustering, structured autoencoders for subspace clustering, and XAI beyond classification: interpretable neural clustering [30,31,32,33] for further models’ improvement and to analyze clustering techniques contribution.

## Figures and Tables

**Table 1:** Model parameters.

Parameter	Description	Value
r	Number of movie genres	20
$n_{1}$	Number of users in the 1st hierarchical level	{50, 100, 150, 200, 250}
$m_{1}$	Number of movies in the 1st hierarchical level	{100, 200, 300, 400, 500}
x	Optimal user’s hierarchical level	2
y	Optimal movie’s hierarchical level	2
β	Tag-based auxiliary regularization parameter	1.7

**Table 2:** The model prediction error with comparisons.

Training Dataset Size (%)	MAE					
	MF	WNMF	F-ALS	BOW-TRSDL	Proposed	
					With Cluster	Deep WNMF
60	0.8859	0.8797	0.8562	0.8363	0.8011	0.8281
80	0.8438	0.8662	0.8315	0.8177	0.7965	0.8101

**Table 3:** Customer cold-start performance.

Cold Start	MAE					
	MF	WNMF	F-ALS	BOW-TRSDL	Proposed	
					With Cluster	Deep WNMF
New 50 users	0.8946	0.8902	0.8954	0.8884	0.8781	0.8908
New 100 users	0.9383	0.9465	0.9472	0.9131	0.9046	0.9165

**Table 4:** Top-10 performance comparisons.

Top-10	Methods					
	MF	WNMF	F-ALS	BOW-TRSDL	Proposed	
					With Cluster	Deep WNMF
Prec@10	0.3247	0.2694	0.2984	0.3392	0.3405	0.3313
Recall@10	0.2053	0.1375	0.1851	0.2113	0.2371	0.2229

## Data Availability

Not applicable.
